# Incidental Discovery of a Duplicated Ovary During Surgery for Acute Appendicitis in an Adolescent

**DOI:** 10.7759/cureus.95140

**Published:** 2025-10-22

**Authors:** Kyriakos Apostolidis, Chrysovalantis Mariorakis, Christos Voskidis, Vasiliki Magaliou, Ioannis Gogoulis, Maria Petridou, Maria Piagkou, George Triantafyllou, Christos Lyrtzis, Ioannis Georgakis

**Affiliations:** 1 Paediatric Surgery Department, Hippokration General Hospital of Thessaloniki, Thessaloniki, GRC; 2 Department of Anatomy, School of Medicine, Faculty of Health Sciences, National and Kapodistrian University of Athens, Athens, GRC; 3 Anatomy and Surgical Anatomy Department, Aristotle University of Thessaloniki, Thessaloniki, GRC

**Keywords:** anatomy, appendicitis, ovarian duplication, surgical anatomy, variation

## Abstract

Acute right lower quadrant abdominal pain in adolescent women commonly raises suspicion for appendicitis; however, the differential diagnosis must also include gynecologic causes. Rare congenital anomalies can complicate the clinical picture and lead to unexpected intraoperative findings. We report the case of a 13-year-old girl presenting with right iliac fossa pain and minimal clinical signs. Laboratory and imaging studies supported a diagnosis of acute appendicitis. During an appendectomy, a duplicated right ovary was incidentally identified - an extremely rare anatomical anomaly with fewer than 30 cases documented in the literature. The appendectomy was completed, the internal genitalia were evaluated and repositioned anatomically, and the patient had an uneventful postoperative course, being discharged within 48 hours. Ovarian duplication is a rare embryologic anomaly that is usually asymptomatic and discovered incidentally. This case highlights the importance of maintaining a broad differential diagnosis in adolescent women presenting with acute abdominal pain and demonstrates the value of systematic intraoperative exploration. It also emphasizes the need for collaboration between pediatric surgeons and gynecologists when such anomalies are encountered. The discovery of rare congenital anomalies during routine surgical procedures underscores the importance of thorough intraoperative assessment and the limitations of preoperative imaging in pediatric emergencies. Documenting such cases adds to the medical literature, enhances awareness of uncommon anomalies, and informs future clinical management.

## Introduction

Acute pain in the right lower quadrant of the abdomen among adolescent women is a common yet diagnostically challenging presentation in both pediatric and emergency medical settings. While acute appendicitis remains one of the most prevalent causes of surgical abdomen in this demographic group, gynecologic etiologies - such as ovarian torsion, ruptured ovarian cyst, pelvic inflammatory disease, or congenital anomalies - must also be considered owing to their overlapping symptoms and proximity of affected structures [1]. Among these rare conditions, ovarian duplication - which encompasses both supernumerary ovaries and accessory ovaries - represents an extraordinarily uncommon congenital anomaly. Its estimated incidence varies from one in 29,000 to 700,000, with fewer than 40 cases of supernumerary ovaries documented in the literature to date [2-4]. Ectopic ovaries are entirely distinct from normal adnexa and possess their own vascular supply, whereas accessory (or supernumerary) ovaries are situated anatomically adjacent and may share structural or vascular connections [2-4]. These anomalies are sometimes associated with other congenital malformations involving the Müllerian ducts or urinary tract [5]. Clinically, they are often asymptomatic and discovered incidentally during imaging or surgical procedures, but may also present with pain, torsion, mass effect, or neoplastic transformation [4,6]. The preoperative diagnosis of ovarian duplication remains notably challenging. Although ultrasonography serves as the initial imaging modality in pediatric and adolescent populations due to its availability and safety, it may lack sufficient resolution to detect such rare anomalies [7]. Even advanced imaging modalities such as magnetic resonance imaging (MRI), which offer higher sensitivity for soft tissue characterization, may misinterpret supernumerary ovarian tissue as atypical cysts or adrenal lesions [4,7]. This underscores the importance of systematic intraoperative assessment, particularly during laparoscopic procedures in female adolescents.

## Case presentation

A 13-year-old girl of Ukrainian descent was transferred from a provincial hospital to the Emergency Department of a tertiary care facility due to progressive right iliac fossa pain that commenced in the morning and intensified throughout the day. The pain was associated with nausea but was not accompanied by fever, vomiting, or bowel disturbances. Her medical history was notable for a COVID-19 infection one month prior, following which she experienced intermittent palpitations and retrosternal tightness.

**Figure 1:**
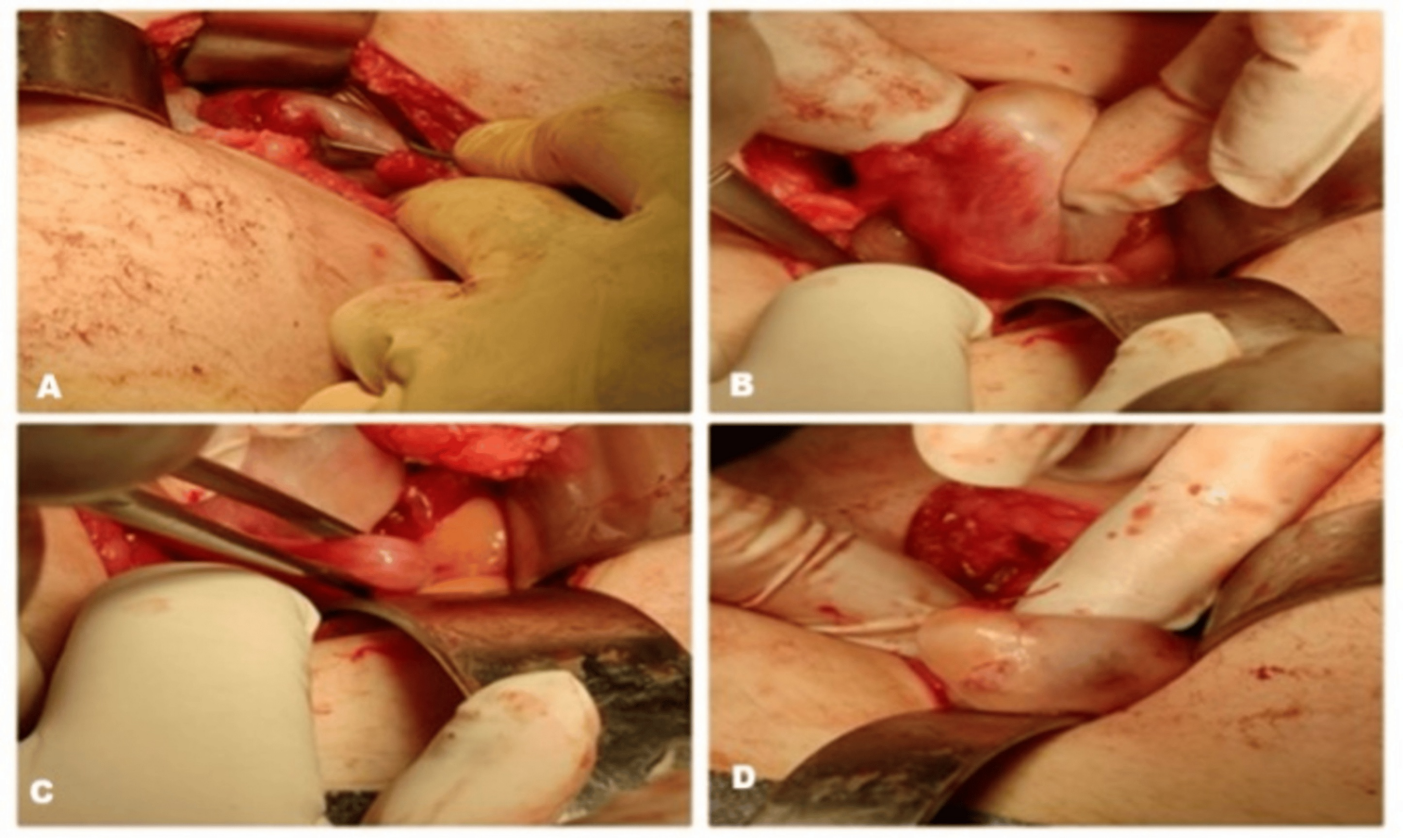
Intraoperative findings of a duplicated right ovary. Intraoperative images demonstrating the discovery of a duplicated right ovary in a 13-year-old girl undergoing appendectomy. (A) Exposure of the pelvic cavity after appendectomy, showing the adnexal region. (B) Identification of an additional adnexal structure adjacent to the right ovary. (C) Careful mobilization of the duplicated ovary using atraumatic forceps. (D) Final inspection confirming the presence of two anatomically distinct right ovaries without evidence of torsion or cystic change.

On examination, the abdomen was soft but tender over the right iliac fossa and lower abdomen. Laboratory investigations revealed mildly elevated inflammatory markers (C-reactive protein (CRP), 1.66 mg/dL) and increased neutrophils (11,200/μL; 81.8%). An abdominal X-ray demonstrated significant aeroplegia, which improved after the administration of glycerin suppositories. However, due to persistent localized tenderness, an abdominal and pelvic ultrasound was performed, which demonstrated findings consistent with acute appendicitis. Given these results, the patient was scheduled for surgery. An open laparoscopy was performed due to the unavailability of the laparoscopic tower during multiple simultaneous emergency cases. Following a routine appendectomy, a systematic intraoperative inspection of the pelvic organs revealed a mass adjacent to the right ovary. Initially suspected to be an ovarian cyst, its location and morphology were atypical. Intraoperative gynecological consultation confirmed the presence of a duplicated right ovary (Figure 1) - a rare anomaly with very limited descriptions in the literature.

The internal genitalia were repositioned into normal anatomical alignment without further intervention, and the procedure was completed. The patient’s postoperative recovery was uneventful, and she was discharged on the second postoperative day. A follow-up examination on the 10th postoperative day showed no abnormalities or complications.

The patient provided informed consent to anonymously publish the data. The research was conducted ethically following the Code of Ethics of the World Medical Association (Declaration of Helsinki).

## Discussion

The differential diagnosis of acute abdominal pain in adolescent women is extensive, encompassing both gastrointestinal and gynecologic conditions. Appendicitis remains the predominant cause of surgical abdomen in this demographic group. Still, gynecologic etiologies such as ovarian torsion, cyst rupture, and pelvic inflammatory disease must also be considered due to their anatomical proximity and overlapping symptomatology [8].

In this case, the clinical findings were most consistent with acute appendicitis, as evidenced by localized tenderness in the right iliac fossa, mildly elevated inflammatory markers, and ultrasonographic signs of appendiceal inflammation. However, the intraoperative discovery of a duplicated right ovary introduced a scarce diagnostic consideration. Ovarian duplication, also known as a supernumerary ovary, is a congenital anomaly with fewer than 40 documented cases worldwide [9]. It is essential to distinguish between ectopic and supernumerary ovaries. An ectopic ovary is entirely separate from the normal adnexa and has its own vascular supply, whereas supernumerary ovary remains structurally or vascularly connected to the native ovary. In our case, the duplicated structure was adjacent but anatomically distinct from the right ovary, suggesting a supernumerary ovary; however, definitive classification would require histological and vascular studies [10-13].

The embryological basis of ovarian duplication remains uncertain; however, the most widely accepted explanation is an abnormal division of the gonadal ridge during embryogenesis, resulting in the formation of additional ovarian tissue. While many cases are asymptomatic and discovered incidentally, complications including torsion, cyst formation, and neoplastic transformation have been described. For instance, Lim et al. [14] reported two concurrent dermoid cysts in an accessory and an ectopic ovary, demonstrating that ectopic ovarian tissue retains malignant potential. Similarly, Zon et al. [15] described a dermoid cyst in an accessory ovary in Thailand, reinforcing this risk.

From a diagnostic standpoint, this case illustrates the limitations of preoperative imaging in identifying rare anomalies. Ultrasound remains the first-line imaging modality in adolescents because of its safety and accessibility, but its resolution is often inadequate for detecting ectopic ovarian tissue. Magnetic resonance imaging (MRI) offers superior soft tissue characterization and, in some reports, has revealed cord-like structures and follicle-like features suggestive of ectopic ovarian tissue. For example, Wang et al. [11] described a fourth supernumerary ovary in the hepatorenal space, which mimicked an adrenal pheochromocytoma on MRI, demonstrating how such anomalies can masquerade as extra-pelvic masses [11,12].

Another consideration is the association between ovarian duplication and congenital anomalies. Zhigang and Wenlu [13] reported an intrarenal supernumerary ovary with concurrent renal pelvis and ureteral duplication, suggesting a shared embryologic pathway for urogenital development. Other studies have described coexisting Müllerian anomalies, endometriosis, and ectopic ovarian tissue in unusual sites such as the rectosigmoid colon [16].

Our patient’s postoperative recovery was uneventful, and the duplicated ovary was left in situ, given the absence of symptoms or complications. However, long-term follow-up is warranted, as the reproductive and gynecological implications remain uncertain [13].

The main strength of this case lies in its documentation of a rare instance of ovarian duplication in an adolescent, thereby contributing to the limited existing literature. It emphasizes the importance of systematic intraoperative exploration in female patients undergoing appendectomy, highlighting the role of interdisciplinary collaboration between pediatric surgeons and gynecologists. An additional strength is the inclusion of a comprehensive literature review, which situates this case within a broader clinical context.

However, several limitations should be acknowledged. As with most case reports, the findings are based on a single patient and therefore lack generalizability. Moreover, histological analysis was not performed, preventing a definitive distinction between a supernumerary and an accessory ovary. The absence of advanced preoperative imaging, such as MRI, limited diagnostic precision, though this reflects the constraints of an emergency surgical setting. Finally, the lack of long-term follow-up data precludes conclusions regarding the patient’s future reproductive and gynecological outcomes.

## Conclusions

This case stresses the importance of maintaining an expansive differential diagnosis when evaluating adolescent patients presenting with acute abdominal pain, as both gastrointestinal and gynecologic causes must be considered. Although imaging is integral to diagnosis, its limitations in identifying rare congenital anomalies such as ovarian duplication highlight the necessity of thorough intraoperative examination. The incidental identification of a duplicated right ovary during an appendectomy underscores the significance of systematic pelvic evaluation in adolescent women and illustrates the critical need for interdisciplinary cooperation between pediatric surgeons and gynecologists. Current literature also suggests that duplicated ovaries may be found in extrapelvic regions, be associated with other congenital anomalies, and possess potential for neoplastic transformation. These observations underscore the importance of diligent intraoperative vigilance and, when appropriate, long-term clinical follow-up. By documenting this case and reviewing existing literature, we contribute to the relatively limited yet expanding body of knowledge concerning ovarian duplication, thereby providing clinicians with additional insights for managing similar rare intraoperative findings.

## References

[1] Bundy DG, Byerley JS, Liles EA, Perrin EM, Katznelson J, Rice HE (2007). Does this child have appendicitis?. JAMA.

[2] Wharton LR (1959). Two cases of supernumerary ovary and one of accessory ovary, with an analysis of previously reported cases. Am J Obstet Gynecol.

[3] Cruikshank S, Van Drie D (1982). Supernumerary ovaries: update and review. Obstet Gynecol.

[4] Lachman MF, Berman MM (1991). The ectopic ovary. A case report and review of the literature. Arch Pathol Lab Med.

[5] Harlass F, Magelssen D, Soisson AP (1987). Supernumerary ovary. A case report. J Reprod Med.

[6] Litos MG, Furara S, Chin K (2003). Supernumerary ovary: a case report and literature review. J Obstet Gynaecol.

[7] Lee B, Gore B (1984). A case of supernumerary ovary. Obstet Gynecol.

[8] Bean JF, Rowell E (2014). Evaluation of the adolescent female with acute lower abdominal pain. Clin Pediatr Emerg Med.

[9] Choudhary N, Farooq O, Dar FA (2023). Study of clinicopathological pattern and outcome of adnexal masses in females from puberty to perimenopause at LD hospital. International Journal of Research and Review.

[10] Woodfield K, Alaniz V, Hutchens K (2024). A supernumerary ovary in an adolescent. J Pediatr Adolesc Gynecol.

[11] Wang Y, Lin Z, Zhou L, Song K, Lu Y, Guan J (2023). The fourth supernumerary ovary in hepatorenal space mimicking an adrenal pheochromocytoma demonstrated with magnetic resonance imaging. Quant Imaging Med Surg.

[12] El-Gohary Y, Pagkratis S, Lee T, Scriven RJ (2015). Supernumerary ovary presenting as a paraduodenal duplication cyst. J Pediatr Surg Case Rep.

[13] Zhigang Z, Wenlu S (2007). An intrarenal supernumerary ovary concurrent with a completely duplicated pelvis and ureter. Int Urogynecol J Pelvic Floor Dysfunct.

[14] Lim MC, Park SJ, Kim SW, Lee BY, Lim JW, Lee JH, Huh CY (2004). Two dermoid cysts developing in an accessory ovary and an eutopic ovary. J Korean Med Sci.

[15] Zon E, Ismail M, Kamaludin Z, Hashim N (2023). Dermoid cyst in an accessory ovary: a case report. Thai Journal of Obstetrics and Gynaecology.

[16] Lim CK, Kim HJ, Pack JS, Ha JG, Yang YS, Lee HK, Kim SH (2018). Supernumerary ovary on recto-sigmoid colon with associated endometriosis. Obstet Gynecol Sci.

